# Length‐dependent MRI of hereditary neuropathy with liability to pressure palsies

**DOI:** 10.1002/acn3.50953

**Published:** 2019-12-24

**Authors:** Michael Pridmore, Ryan Castoro, Megan Simmons McCollum, Hakmook Kang, Jun Li, Richard Dortch

**Affiliations:** ^1^ Vanderbilt University Institute of Imaging Science Vanderbilt University Medical Center Nashville Tennessee USA; ^2^ Department of Neurology Division of Neuromuscular Medicine Wake Forest School of Medicine Winston‐Salem North Carolina USA; ^3^ Department of Neurology Vanderbilt University Medical Center Nashville Tennessee USA; ^4^ Department of Biostatistics Vanderbilt University Nashville Tennessee USA; ^5^ Department of Neurology Wayne State University School of Medicine Detroit Michigan USA; ^6^ Department of Biomedical Engineering Vanderbilt University Nashville Tennessee USA; ^7^ Department of Radiology and Radiological Sciences Vanderbilt University Medical Center Nashville Tennessee USA

## Abstract

**Objective:**

Hereditary neuropathy with liability to pressure palsies (HNPP) is caused by heterozygous deletion of the *peripheral myelin protein 22 (PMP22)* gene. Patients with HNPP present multifocal, reversible sensory/motor deficits due to increased susceptibility to mechanical pressure. Additionally, age‐dependent axonal degeneration is reported. We hypothesize that length‐dependent axonal loss can be revealed by MRI, irrespective of the multifocal phenotype in HNPP.

**Methods:**

Nerve and muscle MRI data were acquired in the proximal and distal leg of patients with HNPP (*n* = 10) and matched controls (*n* = 7). More specifically, nerve magnetization transfer ratios (MTR) were evaluated to assay proximal‐to‐distal gradients in nerve degeneration, while intramuscular fat percentages (*F*_per_) were evaluated to assay muscle fat replacement following denervation. Neurological disabilities were assessed via the Charcot‐Marie‐Tooth neuropathy score (CMTNS) for correlation with MRI.

**Results:**

*F*_per_ values were elevated in HNPP proximal muscle (9.8 ± 2.2%, *P* = 0.01) compared to controls (6.9 ± 1.0%). We observed this same elevation of HNPP distal muscles (10.5 ± 2.5%, *P* < 0.01) relative to controls (6.3 ± 1.1%). Additionally, the amplitude of the proximal‐to‐distal gradient in *F*_per_ was more significant in HNPP patients than controls (*P* < 0.01), suggesting length‐dependent axonal loss. In contrast, nerve MTR values were similar between HNPP subjects (sciatic/tibial nerves = 39.4 ± 2.0/34.2 ± 2.5%) and controls (sciatic/tibial nerves = 37.6 ± 3.8/35.5 ± 1.2%). Proximal muscle *F*_per_ values were related to CMTNS (*r* = 0.69, *P* = 0.03), while distal muscle *F*_per_ and sciatic/tibial nerve MTR values were not related to disability.

**Interpretation:**

Despite the multifocal nature of the HNPP phenotype, muscle *F*_per_ measurements relate to disability and exhibit a proximal‐to‐distal gradient consistent with length‐dependent axonal loss, suggesting that *F*_per_ may be a viable biomarker of disease progression in HNPP.

## Introduction

Hereditary neuropathy with liability to pressure palsies (HNPP) is a dysmyelinating polyneuropathy caused by heterozygous deletion of the *peripheral myelin protein 22* (*PMP22*) gene.^1^ The clinical presentation consists of reversible, focal numbness, and weakness that may or may not be triggered by mechanical compression or repetitive limb movements. Pathological studies reveal “tomacula” (excessively folded myelin in paranodes)^2^ and length‐dependent axonal loss, the latter of which develops during aging.^3^ Previous studies indicate that axonal loss, not demyelination, is correlated with disability in other inherited neuropathies, such as CMT1A.^4^ However, it is still unclear if accumulative axonal loss in HNPP is correlated with disabilities due to the transient and multifocal nature of the disease phenotype.

Although tools are available to assess HNPP (e.g. nerve conduction studies [NCS], skin biopsies, and Charcot‐Marie‐Tooth neuropathy score [CMTNS]^5^), they primarily target distal nerves, which may be degenerated in some patients, resulting in “floor effects.”^6^ Consistent with magnetic resonance imaging (MRI) studies in the brain,^7^, ^8^, ^9^ we demonstrated^10^ that nerve magnetization transfer ratio (MTR) MRI can overcome these limitations and provide sensitive measures to myelin content changes from de/dysmyelination and axonal loss. Others have demonstrated that fat‐water MRI of skeletal muscle assays atrophy and fat replacement in neuromuscular diseases,^11^, ^12^, ^13^, ^14^ which indirectly reflects axonal degeneration.

The present study evaluated these nerve MTR and muscle fat‐water protocols in patients with HNPP. Given that axonal loss may accumulate over time, we hypothesize that nerve/muscle MRI can reveal axonal degeneration in HNPP, thereby serving as a noninvasive, objective measure related to disease severity. Both the proximal and distal leg were scanned to assess length‐dependence. Our results show a correlation between certain MRI findings and disability.

## Methods

### Human subjects and clinical information

Ten patients (100% female, age: 47.1 ± 12.5 years; body mass index, BMI: 29.3 ± 4.3) with genetic confirmation of a *PMP22* heterozygous deletion were recruited from the CMT clinic at the Vanderbilt University Medical Center, which is a part of the Inherited Neuropathy consortium. Seven healthy volunteers (100% female, age: 54.4 ± 3.5 years, BMI: 26.1 ± 6.3) were enrolled as controls by matching for mean age, gender, and BMI (see Table 1).

**Table 1:** Summary demographic and MRI data.

	Controls (*n* = 7)	HNPP (*n* = 10)	*P*‐value
Age (years)	54.4 ± 3.5 (50–59)	47.1 ± 12.5 (18–63)	0.10
BMI (kg/m^2^)	26.1 ± 6.3 (16.95–37.67)	29.3 ± 4.3 (19.79–34.45)	0.19
Female (%)	100	100	–
MTR – sciatic (%)	39.4 ± 2.0 (35.89–41.34)	37.6 ± 3.8 (34.27–45.94)	0.16
MTR – tibial (%)	34.2 ± 2.5 (30.44–36.75)	35.5 ± 1.2 (33.50–37.38)	0.47
Intramuscular fat – proximal (%)	6.9 ± 1.0 (4.89–7.69)	9.8 ± 2.2 (6.71–13.72)	0.01*
Intramuscular fat – distal (%)	6.3 ± 1.1 (5.17–8.09)	10.5 ± 2.5 (6.94–15.14)	<0.001*

Data collected in all subjects within each cohort and presented as mean ± standard deviation (range). *P*‐values from Wilcoxon rank sum analyses are presented, with an asterisk indicating significance. MTR, magnetization transfer ratio; HNPP, hereditary neuropathy with liabilities to pressure palsies.

Clinical disability was assessed via the CMTNS version 2 in all patients. The CMTNS is an ensemble of sensory symptoms, muscle strength, as well as physical findings, with higher scores indicating more impairment (range: 0–36). All HNPP subjects also underwent NCS using conventional methods^15^ (see Table 2). In the control group, subjects self‐reported no symptoms that were suggestive of peripheral nerve diseases and physical examination indicated no evidence of peripheral neuropathies. Due to well‐established normative values in normal controls, it is standard practice that NCS data from patients are evaluated by comparison to these normatives.^16^ As a result, NCS data were not obtained in the control cohort, which did not allow for a correlation of MRI data and clinical findings in this cohort.

**Table 2:** Electrophysiologic findings of HNPP subjects.

	*n*	Mean ± SD	Range	Norm^a^	*P*‐value
CMTNS	10	6.6 ± 3.1	2.0–12.0	–	–
MCV (m/s)					
Median	10	49.30 ± 4.30	44.0–58.0	49	0.91
Ulnar – proximal	10	48.70 ± 5.85	41.0–57.0	49	0.79
Ulnar – distal	8	44.88 ± 10.15	24.0–55.0	–	–
Peroneal – proximal	7	37.29 ± 3.04	32.0–41.0	44	0.02*
Peroneal – distal	7	45.43 ± 8.56	36.0–61.0	–	–
DML (ms)					
Median	10	5.59 ± 1.04	3.9–7.0	4.4	0.01*
Ulnar	10	3.64 ± 0.83	2.9–5.8	3.3	0.28
Peroneal	7	7.21 ± 1.64	5.3–9.7	6.5	0.30
Tibial	9	6.34 ± 2.27	4.6–12.1	–	–
CMAP (mV)					
Median	10	8.50 ± 2.73	3.9–12.0	4	0.004*
Ulnar	10	11.77 ± 7.27	5.2–31.0	6	0.004*
Peroneal	7	2.76 ± 1.16	1.0–4.8	2	0.11
Tibial	9	5.12 ± 3.77	0.8–10.7	–	–
SPL (ms)					
Median	9	4.56 ± 1.08	3.2–6.7	3.5	0.02*
Ulnar	10	3.66 ± 0.57	2.8–4.6	3.5	0.45
Radial	7	2.77 ± 0.27	2.4–3.1	–	–
Sural	5	4.60 ± 0.31	4.2–5.0	4.4	0.31
SNAP (mV)					
Median	9	13.90 ± 7.55	1.1–22.7	22	0.02*
Ulnar	10	15.04 ± 7.90	5.8–34.4	10	0.04*
Radial	7	25.84 ± 13.72	11.0–52.0	–	–
Sural	5	5.84 ± 2.18	3.2–9.2	6	0.88

*P*‐values from Wilcoxon signed rank analyses are presented, with an asterisk indicating significance. HNPP, hereditary neuropathy with liabilities to pressure palsies; CMTNS, Charcot‐Marie‐Tooth neuropathy score; MCV, motor conduction velocity; DML, distal motor latency; CMAP, compound muscle action potential; SPL, sensory peak latency; SNAP, sensory nerve action potential; *n*, number of subjects examined; SD, standard deviation.

a Normative values from Hu et al.^16^ Distal latency numbers listed are the upper limit of normal. Amplitude and conduction velocity numbers are listed in the lower limit of normal. Ulnar and peroneal MCV reported contains both proximal and distal measures.

### Standard protocol approvals, registrations, and patient consents

The study was approved by our local Institutional Review Board and all participants provided informed consent prior to all examinations.

### Data availability

Deidentified data related to the current study will be made available from the corresponding author upon reasonable request.

### MRI data acquisition

**Figure 1:**
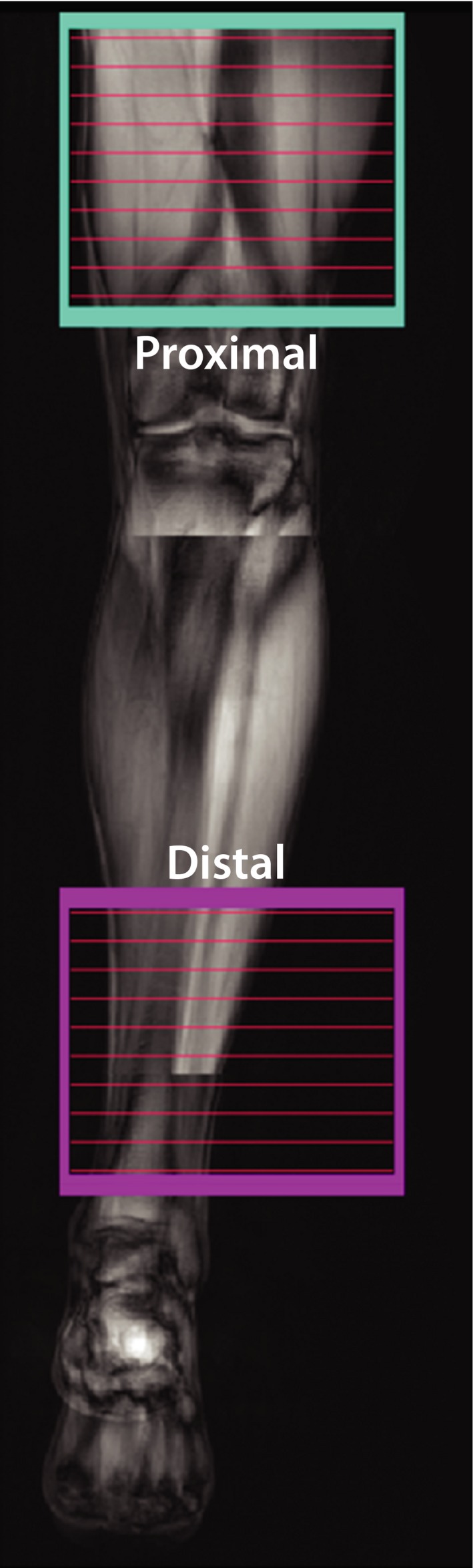
MRI volumes used for leg scan. The proximal leg area is the top, seafoam‐colored box and the distal leg area is the bottom, magenta‐colored box. Red lines inside indicate slice selection.

Participants were examined via MRI, lying feet‐first and supine in a Philips 3.0‐T Acheiva MRI scanner (Philips Healthcare, Best, The Netherlands), with R5.3 software. A Philips dStream 16‐channel anterior coil was used for full coverage of the lower extremities. The multi‐parametric imaging protocol included nerve MTR and muscle fat‐water MRI scans, which were used to estimate nerve MTR and intramuscular fat percentage (*F*_per_), respectively. Two volumes were acquired in each subject as shown in Figure 1. The first volume was acquired proximal to the knee to estimate proximal muscle *F*_per_ and sciatic nerve MTR values. The second volume was acquired distal to the knee to estimate distal muscle *F*_per_ and tibial nerve MTR values.

MTR data were acquired via a 3D, multi‐shot EPI sequence acquired with and without the application of an MT‐prepulse (1000° nominal flip angle, 1.5 kHz off‐resonance) and the following: resolution = 0.8 × 0.8 × 6 mm^3^, TR/TE = 60/11 ms, water‐selective excitation pulse (10°), k‐space lines per shot = 5, SENSE factor = 1, NEX = 2, and a total scan time of ≈6 min. The radiofrequency (RF) transmit field (B_1_) was estimated using the actual flip‐angle imaging approach to correct MTR values for B_1_ variations. Fat‐water data were acquired via a six‐echo gradient echo sequence: resolution = 0.75 × 0.75 × 3 mm^3^, TR/first TE/echo spacing = 300/13/17 ms, and a total scan time of ≈2 min.

### MRI data analysis

B_1_‐corrected MTR maps were estimated using our previously published method,^10^ and mean slice‐wise MTR values were calculated for sciatic/tibial nerves. Nerve MTR was calculated as follows:

$$\text{MTR}=(1-S_{\text{MT}}/S_{\text{REF}})\times 100\%,$$

where S_MT_ represents the signal intensities of the MT‐weighted volume and *S*_REF_ represents the signal intensities in the reference (no MT‐weighting) volume. For MTR measurements, slice‐wise regions‐of‐interest (ROIs) were manually selected for the sciatic (proximal volumes) and tibial nerves (distal volume) using in‐house written software in MATLAB (Mathworks, Natick, MA, version 2017a).^17^, ^18^ These ROIs were then multiplied by a mask from MIPAV's (NIH, Bethesda, MD) fuzzy C‐means segmentation algorithm, which eliminated background voxels as well as voxels that had been partially volume averaged with fat, and mean slice‐wise MTR values were estimated.

The ratio of the fat‐to‐water signal, or fat percentage (*F*_per_), was estimated from the multi‐echo gradient data via the optimized tools within the ISMRM Fat‐Water Toolbox.^19^ From these maps, the entire muscle volume was segmented from surrounding fat and the median *F*_per_ was tabulated to ensure that the reported value was indicative of intramuscular fat replacement.

### Statistical analysis

All statistical analyses were conducted using MATLAB. Tests were performed to evaluate (1) differences between patients and controls for each MRI measure, (2) within‐cohort differences between scanning locations (proximal vs. distal) for each MRI measure, (3) across‐cohort differences in the strength of the effect of scanning locations (distal minus proximal) for each MRI measure (i.e. tests for whether length‐dependent effects were more significant in patients), and (4) the relationship between MRI measures and clinical disability score (CMTNS) in patients. Nonparametric statistical approaches were primarily employed for analysis due to the small sample size of the study. Given the exploratory nature of this study, we did not correct for multiple comparisons and deemed a threshold of *P* < 0.05 as indicative of relationships that warrant further investigation.

Four MRI parameters were included in these analyses: sciatic nerve MTR, tibial nerve MTR, proximal muscle *F*_per_, and distal muscle *F*_per_. We first tested for differences in each MRI parameters across cohorts with a Wilcoxon rank sum test. In addition, Wilcoxon signed rank tests were employed within each cohort to test for length‐dependent differences in each MRI parameter per subject (proximal and distal leg measurements). The difference between proximal and distal measures was then estimated for each subject and MRI measure, and linear regression analyses were performed across cohorts to test for significant inter‐cohort differences in these length‐dependent effects. Finally, associations between MRI parameters and CMTNS were tested via Spearman's rank correlations.

Although we attempted to match our control and HNPP cohorts for age and BMI, subtle differences between cohorts may influence findings. As a result, we tested for (1) differences in age and BMI across cohorts using a Wilcoxon rank sum test and (2) associations between the MRI parameters and both age and BMI in our control cohort using Spearman's rank correlations. In addition, NCS values in patients with HNPP were compared to normative values using a Wilcoxon signed rank test.

## Results

### Clinical features of patients

Significant differences for age (*P* = 0.10) and BMI (*P* = 0.19) were not detected across the control and HNPP cohorts. In addition, significant relationships were not observed between BMI (*P* > 0.12) and age (*P* > 0.43) and any of the reported MRI parameters.

All patients with HNPP reported 1–3 incidents of reversible focal sensory loss and weakness throughout their life, which may or may not be triggered by mechanical compression. Results of NCS show focally slowed nerve conduction at the sites susceptible to mechanical pressure. For instance, eight out of 10 patients had a significantly prolonged distal latency of the median motor nerve across the wrist (average value in Table 2 = 5.59 ± 1.04 ms), but only two out of 10 patients had a mildly prolonged distal latency of ulnar motor nerve across the wrist (average value in Table 2 = 3.64 ± 0.83). A similar difference was observed between peroneal and tibial motor nerves. In addition, a focal slowing of conduction velocities was observed in the (1) ulnar motor nerves across the elbow in two patients and (2) peroneal motor nerve across the fibular head in the other two patients. All these features suggest a representative population typical of the HNPP phenotype.^20^, ^21^

In addition, only one patient had a reduced amplitude of sensory nerve action potential (SNAP) in the ulnar nerves, but sural nerves in all patients were either nonresponsive or had a reduced amplitude of SNAP.

### Muscle *F*_per_ values are elevated in HNPP

**Figure 2:**
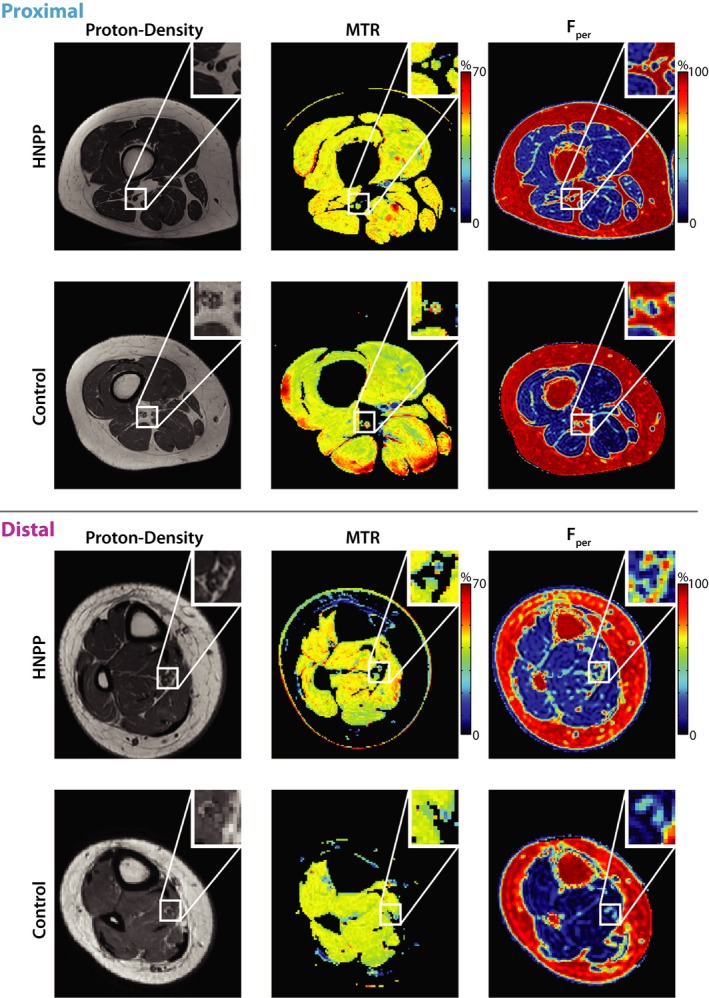
Proximal: Sample results of proton density (left), MTR (middle), and fat‐water (right) imaging in proximal leg (thigh) for a representative HNPP patient (top row) and control subject (bottom row). Each imaging result includes a zoomed‐in detail (top‐right) showing the location of the sciatic nerve. Distal: Sample results of proton density (left), MTR (middle), and fat‐water (right) imaging in distal leg (ankle) for a representative HNPP patient (top row) and control subject (bottom row). Each imaging result includes a zoomed‐in detail (top‐right) showing the location of the tibial nerve. MTR, magnetization transfer ratio; HNPP, hereditary neuropathy with liabilities to pressure palsies.

**Figure 3:**
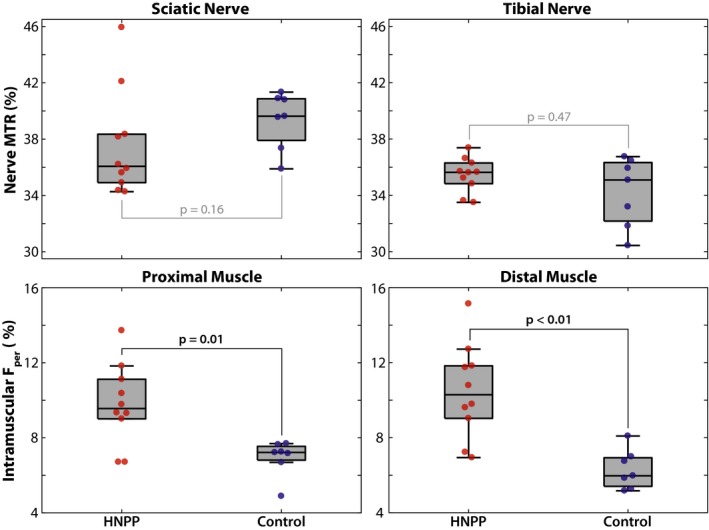
Differences between groups per MRI measure. Sciatic/tibial nerve MTR and proximal/distal leg *F*_per_ values were compared across HNPP and control groups. For the boxplots, the central mark is the median, the edges of the box are the 25th and 75th percentiles, and the whiskers extend to all data points not deemed outliers. Corresponding data from individual patients with HNPP (red) and controls subjects (blue) are overlaid. Significant differences across groups are indicated with black text, while nonsignificant differences are indicated with gray text. MTR, magnetization transfer ratio; HNPP, hereditary neuropathy with liabilities to pressure palsies.

Figure 2 shows sample MRI findings in the proximal and distal leg, respectively, and these results are further quantified for each cohort in Figure 3 and Table 1. As shown in the zoomed insets, the sciatic and tibial nerves can be readily distinguished from surrounding fat (appears bright in the proton density images), muscle, and blood vessels in all slices. Across cohorts, *F*_per_ values in patients with HNPP were significantly elevated in proximal (*P* = 0.01) and distal muscles (*P* < 0.01) relative to controls. In contrast, MTR values were similar across cohorts in both the sciatic nerve (*P* = 0.16) and tibial nerve (*P* = 0.48).

### Muscle *F*_per_ values exhibit length‐dependent changes

**Figure 4:**
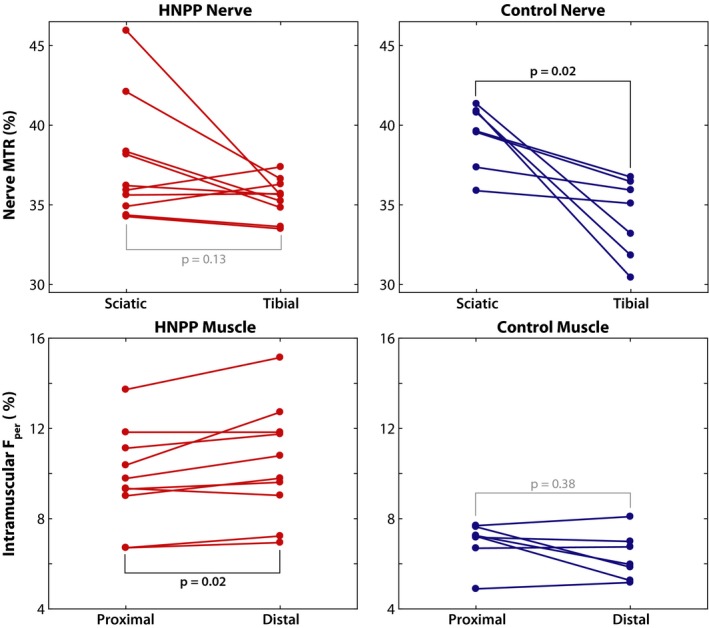
Differences between scanning locations per group. HNPP (red) and control (blue) MRI measures (MTR and *F*_per_) were compared across scanning locations (proximal and distal). Each line represents the difference that occurred in a single subject between both scanning locations. Significant differences across locations are indicated with black text, while nonsignificant differences are indicated with gray text. MTR, magnetization transfer ratio; HNPP, hereditary neuropathy with liabilities to pressure palsies.

Paired MRI results between proximal and distal tissues per group and MRI metric are shown in Figure 4. A significant increase in distal *F*_per_ values was detected in HNPP patients (proximal/distal muscle = 9.8 ± 2.2/10.5 ± 2.5%, *P* = 0.02), but not controls (proximal/distal muscle = 6.9 ± 1.0/6.3 ± 1.1%, *P* = 0.38), which is consistent with the length‐dependent nature of axonal loss. Furthermore, linear regression of the difference between proximal and distal measures across cohorts showed that HNPP patients exhibited significantly stronger length‐dependent changes compared to controls (*P* = 0.01, adjusted *R* ^2^ = 0.35).

In contrast, sciatic and tibial nerve MTR values were similar in HNPP subjects (sciatic/tibial nerves = 39.4 ± 2.0/34.2 ± 2.5%, *P* = 0.13). In addition, although controls (sciatic/tibial nerves = 37.6 ± 3.8/35.5 ± 1.2%, *P* = 0.02) showed significantly lower values in distal tibial nerves, linear regression of the difference between proximal and distal measures across cohorts did not indicate that this affect was stronger than that observed in patients (*P* = 0.13, adjusted *R* ^2^ = 0.09).

### Muscle *F*_per_ values relate to disability

**Figure 5:**
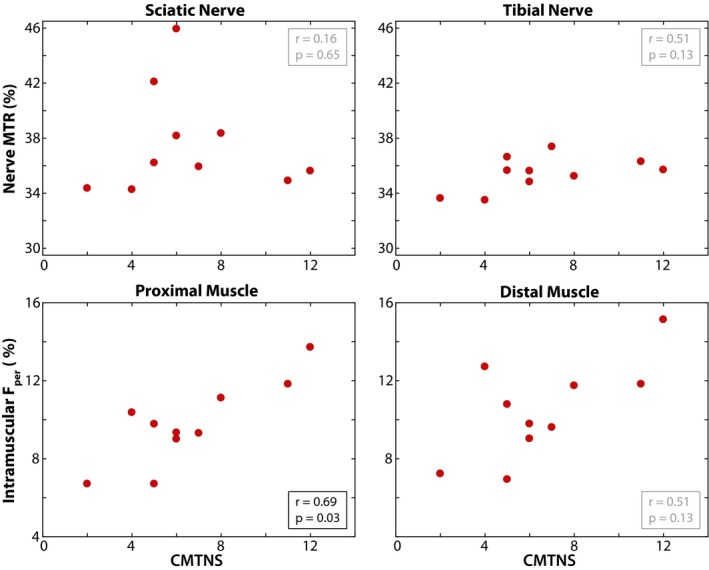
MRI measures in patients with HNPP relate to clinical neuropathy score. Sciatic/tibial nerve MTR and proximal/distal muscle *F*_per_ measures were correlated to CMTNS scores in the patient group. Significant relationships between MRI measures and CMTNS are indicated with black text, while nonsignificant relationships are indicated with gray text. MTR, magnetization transfer ratio; HNPP, hereditary neuropathy with liabilities to pressure palsies; CMTNS, Charcot‐Marie‐Tooth neuropathy score.

As shown in Figure 5, a significant relationship was observed between proximal muscle *F*_per_ and CMTNS measures (*r* = 0.69, *P* = 0.03). Sciatic nerve MTR (*r* = 0.16, *P* = 0.65), tibial nerve MTR (*r* = 0.51, *P* = 0.13), and distal muscle *F*_per_ (*r* = 0.51, *P* = 0.13) were not significantly related to CMTNS.

## Discussion

In this study, we demonstrated that muscle *F*_per_ values are elevated in patients with HNPP. The changes in *F*_per_ support a length‐dependent process of denervation that results from axonal loss in the disease. Therefore, despite the multifocal nature of phenotype in HNPP, pathology accumulated over time still results in length‐dependent axonal degeneration, which is consistent with previous reports in animal models^20^ and human studies in older subjects.^22^ In line with previous literature in other types of neuropathies,^23^ this axonopathy in HNPP appears to be the key factor leading to disabilities since *F*_per_ value is correlated with CMTNS.

Although numerous biomarkers have been proposed for CMT subtypes (NCS,^24^ quality of life assessments, sensorimotor testing, CMTNS), they may be compromised in HNPP due to the disease phenotype. For example, although quality of life is affected in HNPP, these surveys are not particularly sensitive to disease progression in inherited neuropathies.^25^, ^26^ In addition, CMTNS may be inadequate to describe the progression of HNPP given the transient nature of clinical deficits. For instance, proximal arm weakness by brachial plexopathy may result in a high score of CMTNS, but only lasts a few weeks. Once the weakness recovers, CMTNS would decrease substantially, presenting difficulties in assessing disease progression.

In contrast, MRI can be used as a noninvasive, quantitative, and objective tool to simultaneously assess pathologies in the proximal and distal nerves and muscles of patients with HNPP. In other words, the quantitative MRI methods presented herein may improve our ability to longitudinally study HNPP, provide viable biomarkers for disease progression and treatment response independent of the clinical presentation of HNPP, and may provide insight into the pathogenesis of the disease.

Fat‐water MRI exploits the chemical shift difference inherent in fat and water protons in order to generate fat percentage maps of tissue.^14^, ^27^, ^28^ Developments in skeletal muscle MRI methods^29^, ^30^ have allowed researchers to assay the possible downstream effects of denervation in other inherited neuropathies such as Charcot‐Marie‐Tooth neuropathy type 1A (CMT1A).^11^ Fat‐water measurements have been shown to be valid, reliable,^31^ and sensitive to CMT1A progression,^11^ making this method a viable option for use as a disease biomarker for HNPP as they arise from abnormalities in the *PMP22* gene.^1^ However, previous studies have focused on single‐slice acquisitions. Here, we applied this method to HNPP patients for the first time at multiple levels of limbs. To better assess the relationship between length‐dependent axonal loss and disease progression, we performed MRI in the proximal and distal leg, noting that the optimal level of deployment for these measurements may vary across patients and time due to the length‐dependent nature of CMT. In this study, we found (1) *F*_per_ measures were elevated in HNPP patients relative to controls consistent with previously published findings,^11^ (2) the observed elevation was significantly higher in the distal leg muscles consistent with the length‐dependent nature of this disease, and (3) *F*_per_ measures were related to CMTNS disability scores.

One potential drawback of these findings is that fat replacement represents the downstream effect of the nerve pathology and is the chronic endpoint of disease progression and, thus, is unlikely to be reversed with treatment. Additionally, the method only gives us information on motor fibers, and muscle atrophy patterns are not consistent between patients.^32^ It should also be noted that these differences may be influenced by factors not associated with the disease process, such as the degree and frequency of exercise.^33^

As a result, we deployed our previously developed nerve MTR protocol to overcome these limitations and directly assay peripheral neuropathy in the nerves of the leg. Previous studies have shown that MTR correlates with myelin content changes^34^, ^35^, ^36^ that result from de/dysmyelination and axonal loss in the central and peripheral nervous system.^37^ Furthermore, previous work has indicated that nerve MTR (1) is reliable and related to disability across multiple cohorts with inherited neuropathies^10^ and (2) exhibits proximal‐to‐distal gradient in controls that increases with age, which is consistent with the findings from the older control cohort studied herein (Figure 4, upper‐right panel).^40^ In the current study, we measured proximal nerve MTR in HNPP for the first time and used this method in distal leg nerves to probe length‐dependent effects. We found that, surprisingly, MTR was similar in controls and patients with HNPP. We postulate that these findings are due to the competing effects of axonal loss, which reduces MTR values, and the formation of tomacula, which is expected to increase MTR values. This is consistent with previous ex vivo nerve studies in mouse models of HNPP, where similar MT effects were observed in the sciatic nerve of mice with PMP22 deletions and age‐matched wild‐type mice.^38^ The MTR findings herein are consistent with these ex vivo studies and are supportive of a length‐dependent axonal loss superimposed on the aforementioned focal abnormalities in HNPP. When axons degenerate, denervated muscles become atrophic with a fraction of muscles replaced by fat tissues. Thus, muscle *F*_per_ by MRI provides complementray, albeit indirect, information on axonal loss.^39^

Despite promising results, the current study had several limitations. Most notable of these limitations is the sample size. HNPP is a rare disease with an estimated prevalence of 2–5:100,000, although the actual prevalence may be underestimated.^5^ The second main limitation is the cross‐sectional design of this study. Testing the relationship of BMI and age with our MRI parameters suggests that the observed differences in MRI parameters across cohorts are not confounded by these demographic factors. Nevertheless, longitudinal studies are warranted to investigate the responsiveness of each metric to progression over time. Finally, MTR is only a semiquantitative measure as it is difficult to implement and is sensitive to differences in hardware. For example, although we attempted to correct for B_1_ errors, our previous results^10^ showed that this can be a source of variance. This can be further visualized in the superficial muscle regions in the control MTR map shown in Figure 2, where MTR was overestimated due to large B_1_ errors within these regions (red hues). Although we did not observe similar errors within the nerves themselves, this could partially explain why we did not observe a significant relationship between MTR and CMTNS in our analysis. Future work will focus on quantitative MT methods that can overcome these issues and provide more reliable and specific measures of myelin content.^41^ In addition, the novel contrast from the MTR sequence may also be useful in other more common neuropathies and/or aid in the anatomical segmentation of nerves.^42^

In conclusion, this study demonstrates the importance of implementing multi‐parametric acquisitions (MTR/*F*_per_) at multiple scanning locations (proximal/distal leg) to assess HNPP. Our measurements show that (1) fat percentage is elevated in the HNPP population, (2) there are length‐dependent changes of fat percentage occurring between the proximal and distal leg locations, and (3) fat percentage in proximal muscle correlates with clinical disability in patients with HNPP. Our MTR measures did not detect significant differences between cohorts, and this may be due to the fact that (1) our HNPP sample size is small and (2) MTR values are affected by both myelin content and the formation of tomacula – both of which are occurring in HNPP. These results herein suggest that these measures, particularly muscle fat percentages, may be of value as a biomarker for HNPP disease and warrant further investigation in larger longitudinal studies.

## Author Contributions

M.P.: drafting/revising the manuscript, study concept and design, analysis and interpretation of data, acquisition of data, and statistical analysis; R.C.: acquisition of data and subject recruitment; M.S.M.: drafting/revising the manuscript, acquisition of data, and subject recruitment; H.K.: drafting/revising the manuscript and statistical analysis; J.L.: drafting/revising the manuscript, study concept and design, interpretation of data, acquisition of data, funding support, and study supervision; R.D.: drafting/revising the manuscript, study concept and design, interpretation of data, acquisition of data, funding support, and study supervision.

## Conflict of Interest

No authors in this manuscript have any conflict of interest to disclose.
